# Disruption of zinc homeostasis reverses tigecycline resistance in *Klebsiella pneumoniae*

**DOI:** 10.3389/fcimb.2025.1458945

**Published:** 2025-02-12

**Authors:** Jinyu Wang, Cuiping Xia, Zhaoxin Xia, Jilu Shen

**Affiliations:** ^1^ The First Affiliated Hospital of Anhui Medical University, Clinical laboratory, Hefei, Anhui, China; ^2^ Anhui Public Health Clinical Center, Clinical laboratory, Hefei, Anhui, China

**Keywords:** PBT2, zinc, GlmU, tigecycline, Klebsiella pneumoniae

## Abstract

**Introduction:**

Zinc exhibits potent antimicrobial properties due to its ability to compromise bacterial structure and protein functionality, effectively inhibiting and eradicating bacteria. However, bacteria have evolved mechanisms to expel excess zinc ions from their cells, enabling them to thrive in environments rich in metal ions at high concentrations. This evolutionary advancement limits the clinical application of metal ions as antimicrobial agents. In this study, we aimed to investigate the potential of zinc ionophores to overcome bacterial resistance by elevating intracellular zinc ion levels.

**Methods:**

We employed the zinc ionophore PBT2 to elevate intracellular zinc ion levels in Klebsiella pneumoniae, a bacterium known for its resistance to various antibiotics. By treating K. pneumoniae with PBT2, we aimed to assess its impact on bacterial resistance to tigecycline, an antibiotic commonly used in clinical settings. The changes in intracellular zinc ion levels, superoxide dismutase activity, reactive oxygen species concentration, and cell wall synthesis pathway were monitored to evaluate the mechanism of action of PBT2.

**Results:**

Our results revealed that PBT2 successfully reversed the resistance of K. pneumoniae to tigecycline. Specifically, PBT2 increased the concentration of intracellular zinc ions in K. pneumoniae, leading to a suppression of superoxide dismutase activity within the cell and an elevation of reactive oxygen species concentration. These changes impaired the oxidative stress response of the bacteria. Additionally, the disruption of zinc homeostasis significantly inhibited the cell wall synthesis pathway in K. pneumoniae, potentially restricting the efflux pump mechanism that predominantly drives tigecycline resistance.

**Discussion:**

The findings of this study pave the way for innovative strategies and approaches in the clinical development of novel antimicrobial agents. By using zinc ionophores such as PBT2 to elevate intracellular zinc ion levels, we can overcome bacterial resistance to antibiotics like tigecycline. The suppression of superoxide dismutase activity and elevation of reactive oxygen species concentration suggest that PBT2 impairs the oxidative stress response of K. pneumoniae, further enhancing its susceptibility to antibiotics. Furthermore, the inhibition of the cell wall synthesis pathway and restriction of the efflux pump mechanism provide additional mechanisms by which PBT2 reverses antibiotic resistance. These results highlight the potential of zinc ionophores as a novel class of antimicrobial agents and warrant further investigation into their clinical applications.

## 1 Introduction

Globally, bacterial resistance due to the misuse and inappropriate use of antimicrobial drugs has emerged as a significant public health concern (Samreen et al., 2021; Mendelson et al., 2022). Moreover, the slowdown in the discovery and commercialization of new antimicrobial drugs further exacerbates this issue. Researchers are actively exploring various strategies to address bacterial resistance to existing antimicrobial drugs. These strategies include the development of new antibiotics, phage therapy, and repurposing existing drugs (Wang et al., 2020; Donlin and Meyers, 2022). Some studies suggest that repurposing existing drugs may be a promising strategy. These drugs may not have been initially developed as treatments for infectious diseases. Rediscovering the potential of these drugs and adjusting their existing therapeutic uses can reduce the time, cost, and risk associated with developing new drugs, providing a novel approach to tackling antibiotic resistance (De Oliveira et al., 2020; De Oliveira et al., 2022b).

5,7-Dichloro-2-[(dimethylamino)methyl]quinolin-8-ol (PBT2) has previously been investigated as a potential treatment for Alzheimer’s and Huntington’s diseases and has entered Phase II clinical trials. Experimental results have shown that PBT2 has the ability to facilitate the intracellular transport of zinc ions. PBT2 is a second-generation hydroxyquinoline analogue. As a metal ion carrier, PBT2 has been shown to bind zinc ions in a 2:1 ratio, and the formed PBT2-zinc complex can transport tightly regulated zinc ions into bacterial cells. Zinc is the second most abundant transition metal ion in the human body and plays a crucial role in immune function. According to relevant literature reports, zinc deficiency affects many people worldwide, resulting in adverse effects on human health, including compromised immune defenses and increased susceptibility to infections (Guilbert, 2003; Haase and Rink, 2014a; Haase and Rink, 2014b). Bacterial pathogens encounter significant fluctuations in metal ion abundance during host colonization. Related research has confirmed that zinc contributes to resisting bacterial infections (Maunders et al., 2024; Stiles et al., 2024; Wang et al., 2024), but the specific mechanisms are still unclear. To better understand and address bacterial resistance issues, further research is needed on the effects of zinc stress on bacterial resistance mechanisms. This can lead to a better understanding of bacterial resistance mechanisms against antibiotics and the exploration of more effective antibacterial treatment methods.

Carbapenem-resistant *Klebsiella pneumoniae* (CRKP) is one of the important nosocomial pathogens (Karampatakis et al., 2023), capable of causing various infections and developing resistance to multiple antimicrobial drugs (Kumar et al., 2023; Li et al., 2023). The emergence of multidrug resistance limits the choice of available drugs, with tigecycline and polymyxins becoming the last resort. Tigecycline belongs to the glycylcycline class of antibiotics, which inhibits bacterial protein synthesis by binding to the 16S rRNA of the 30S ribosomal subunit, overcoming the resistance mechanisms of acquired ribosomal protection and active efflux associated with tetracycline drugs, reducing the likelihood of bacterial resistance to tigecycline (Liu et al., 2024). Among these mechanisms, the efflux pump transport system plays a crucial role in resistance. Efflux pumps are widely present on bacterial cell membranes, allowing bacteria to pump antimicrobial drugs entering the cell out of the cell, thereby reducing the drug concentration inside the bacterial cell and leading to resistance. In *Klebsiella pneumoniae*, there are various efflux pump systems, such as the AcrAB-TolC efflux pump, OqxAB efflux pump, and KpgABC efflux pump, among others. Overexpression of these efflux pumps can mediate tigecycline resistance (Yang et al., 2023; Zheng et al., 2023). In this study, we found that the combined use of PBT2 and zinc ions can reverse tigecycline resistance in carbapenem-resistant *Klebsiella pneumoniae*. This mechanism differs from traditional forms of drug resistance, as the antimicrobial action of metal ions makes it difficult for bacteria to develop corresponding resistance.

## 2 Results

### 2.1 Strain selection

In this experiment, a total of 14 strains of tigecycline-nonsusceptible *Klebsiella pneumoniae* (TNSKP) were collected. The drug susceptibility tests revealed that the MIC of tigecycline ranged from 4 to 32 mg/l (**Supplementary Table S1**). Co-incubation with the efflux pump inhibitor PaβN resulted in a ≥4-fold decrease in the MIC values of 14 TNSKP strains, confirming the involvement of efflux pumps in tigecycline resistance.

### 2.2 The effect of PBT2 combined with zinc on the growth of *Klebsiella pneumoniae*

The KP29 strain exhibited an eightfold decrease in MIC value following co-incubation with the efflux pump inhibitor, further confirming that tigecycline resistance is primarily mediated by efflux pumps. Based on these results, the KP29 strain was selected for further investigation. We chose the subinhibitory concentration of PBT 2 (64 μM) as the drug concentration for the subsequent study. We explored the effects of CA-MH broth (MH), zinc ion group (Zinc), PBT2 group (PBT2), and PBT2+Zinc ion group (PZinc) on the alteration of multidrug resistance in *Klebsiella pneumoniae*. Under conditions of PBT2 (64μM) and zinc ions (256μM), the MIC for tigecycline in *Klebsiella pneumoniae* changed by more than fourfold (**Table 1**). Growth curve experiments showed no significant changes with the sole addition of Zinc (256μM), whereas the addition of PBT2 (64μM) alone significantly altered the growth curve. The most pronounced changes were observed when both PBT2 (64μM) and Zinc (256μM) were added together (**Supplementary Figure S1**). This suggests that the combination of PBT2 and Zinc exerts the most effective inhibition on bacterial growth.

**Table 1:** PBT 2 combined with Zinc resensitized K. pneumoniae to tigecycline.

Antibiotic	MIC			
	PBT2:0µ M, Zinc:OµM	PBT2:64µM, Zinc:256µM	PBT2:64µM, Zinc:OµM	PBT2:0µM, Zinc:256µM
Imipenem	64	32	64	64
Meropenem	64	32	64	64
Ampicillin	>128	>128	>128	>128
Doxycycline	32	8	8	32
Tigecycline	16	2	8	16
Minocycline	32	2	8	32
Ceftazidime	>128	>128	>128	>128
Gentamicin	>128	>128	>128	>128

Data represents mean of 3 biological replicates.

### 2.3 Results of metal ion concentration detection and SOD enzyme activity detection

**Figure 1:**
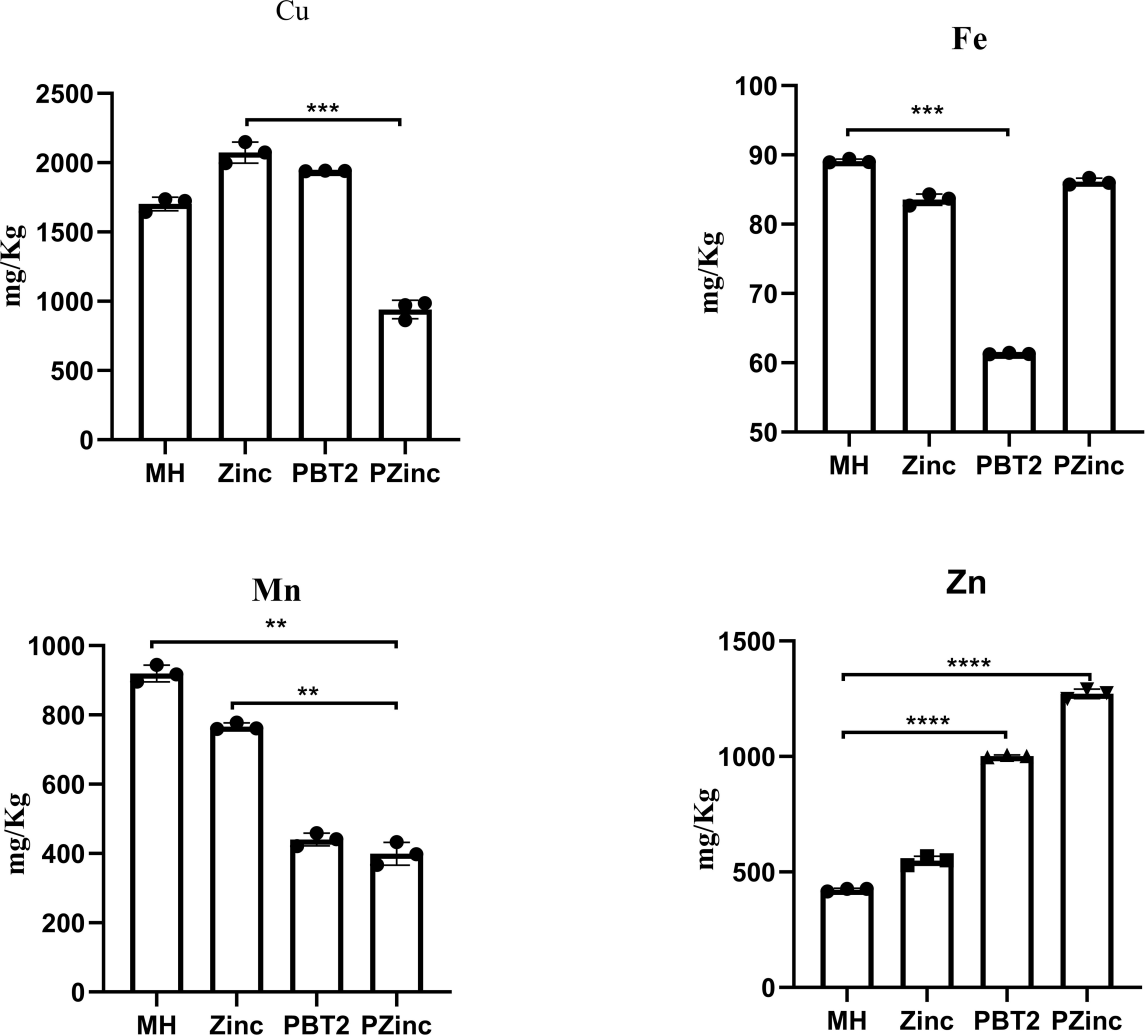
Altered intracellular ion concentrations of K. pneumoniae in different environments. Error bars represent the standard deviations of the means from biological triplicates. **, P < 0.005; ***, P < 0.001; ****, P < 0.0001 by one-way analysis of variance (ANOVA).

**Figure 2:**
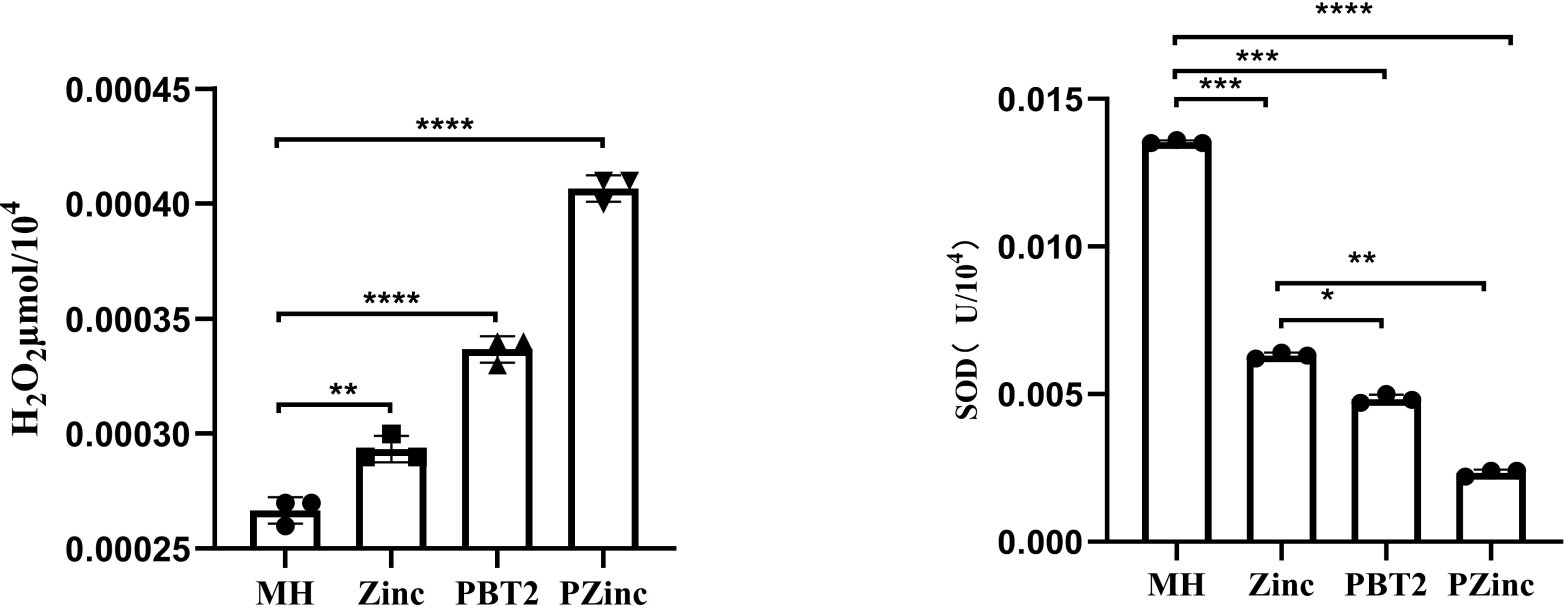
Detection of H2O2 content and SOD enzyme activity in K. pneumoniae under different conditions. Error bars represent the standard deviations of the means from biological triplicates. *, P < 0.05; **, P < 0.005; ***, P < 0.001; ****, P < 0.0001 by one-way analysis of variance (ANOVA).

To further ascertain the factors influencing the change in tigecycline MIC values, we examined the metal ion concentrations in KP29 using Inductively Coupled Plasma Mass Spectrometry (ICP-MS). Detection revealed a decrease in copper ion concentration and a significant increase in zinc ion concentration in the PZinc group. Notably, the sole addition of zinc ions did not markedly alter the intracellular zinc ion concentration, whereas the addition of PBT2 alone significantly enhanced it (**Figure 1**). Concurrently, there was an decrease in the concentration of manganese ions within the cells. In bacterial oxidative stress systems, manganese plays a role in combating oxidative damage. Manganese ions, as a cofactor for SOD enzymes, are crucial for managing reactive oxygen species. Consequently, this study measured the levels of intracellular hydrogen peroxide (H2O2) and superoxide dismutase (SOD) in *Klebsiella pneumoniae*. The results indicated a significant decrease in SOD activity and a marked increase in intracellular H2O2 concentration in the PZinc group compared to the MH group (**Figure 2**).

### 2.4 Drug resistance development experiment

We then explored the potential for resistance development in the KP29 strain under the influence of PBT2 and zinc ions. Sub-inhibitory concentrations of PBT2 and zinc ions were combined with tigecycline to induce resistance over a 30-day period, with doxycycline serving as the control group. The induced resistance experiment results indicated a 64-fold change in resistance to doxycycline in the control group, while the resistance to tigecycline in the experimental group remained unchanged (**Supplementary Figure S2**). The findings suggest that the influx of zinc ions mediated by PBT2 affected the resistance to tigecycline. Unlike traditional pathways of resistance development, the changes in resistance caused by this intracellular high-zinc environment are difficult to reverse.

### 2.5 Results of the metabolomics experiments and electron microscopic scanning experiments

Metabolomics experimental results suggest that zinc stress appears to have affected the biosynthesis of peptidoglycan (**Supplementary Figure S3**). Liquid chromatography-tandem mass spectrometry (LC-MS) results showed a significant reduction in the accumulation of UDP-N-acetylglucosamine (UDP-GlcNAc), yet the expression of the key enzyme in peptidoglycan synthesis, N-acetylglucosamine-1-phosphate uridylyltransferase (GlmU), was upregulated (2.9log2-fold, **Table 2**). These experimental results confirm that zinc enrichment did not affect the expression of the GlmU gene but may have interfered with the function of the GlmU protein to some extent, hindering cell wall synthesis. Building on this, we further observed the impact of PBT2 on the cellular morphology of *Klebsiella pneumoniae*. Scanning electron microscopy revealed that in the PBT2+Zinc group, the morphology of KP bacteria underwent significant changes, with the cell wall showing noticeable shrinkage and collapse (**Supplementary Figure S4**).

**Table 2:** Results of expression changes of several key genes in the PZinc group.

Gene id	MeanTPM (PZinc)	MeanTPM (MH)	log2FoldCha nge	p Value	q Value	Result
Oqxb	316.185	35.5862	3.151379136	0	0	up
AcrB	128.669	20.9765	2.616818625	0	0	up
RND	42.3417	1.0966	5.27096991	0	0	up
AcrD	25.5798	0.925278	4.788974285	4.77E-130	2.46E-129	up
GT2_GlmU_N_bac	152.011	23.568	2.689274485	0	0	up
LbH_UDP-GlcNAc_AT	114.414	35.6732	1.681351054	3.51E-55	l.l OE-54	up
UDPNAET	60.9356	9.6915	2.65249345	l .36E-34	3.54E-34	up
FosA	53.9529	2.46454	4.452310206	2.14E-22	4.73E-22	up
GlmM	44.2104	10.8815	2.022508343	l.13E-19	2.37E-19	up

Oqxb, AcrB, RND, and AcrD are genes related to bacterial efflux pumps, and their expression is upregulated. GT2-GlmU-N-bac, LbH-UDP-GlcNAc-AT, UDPNAET, FosA, and GlmM are key genes involved in bacterial cell wall synthesis, and their expression is also upregulated.

### 2.6 Results of the transcriptomics experiments

To further investigate the impact of intracellular zinc accumulation mediated by PBT2 on *Klebsiella pneumoniae*, we conducted whole-genome sequencing and transcriptome sequencing on the KP29 strain. Through whole-genome sequencing, we extensively examined the situation of the AcrB and Oqxb efflux pump genes. The results showed that under zinc stress conditions, these efflux pump genes did not undergo genetic mutations. This ruled out gene mutation as a reason for zinc ion affecting resistance. Subsequently, we performed transcriptome sequencing to observe the expression changes of these efflux pump genes. Interestingly, in the PBT2+Zinc treatment group, the expression of AcrB and Oqxb efflux pump genes was upregulated (3.2log2-fold, 2.6log2-fold). However, in the PBT2 or Zinc treatment groups alone, there were no significant changes in the expression of these genes (**Table 2**). Combined with the previous MIC experimental results (**Table 1**), we inferred that the phenomenon of resistance reversal caused by zinc ion influx was not due to transcriptional inhibition of efflux pump genes.

### 2.7 Construction of the efflux pump gene knockout strains

**Figure 3:**
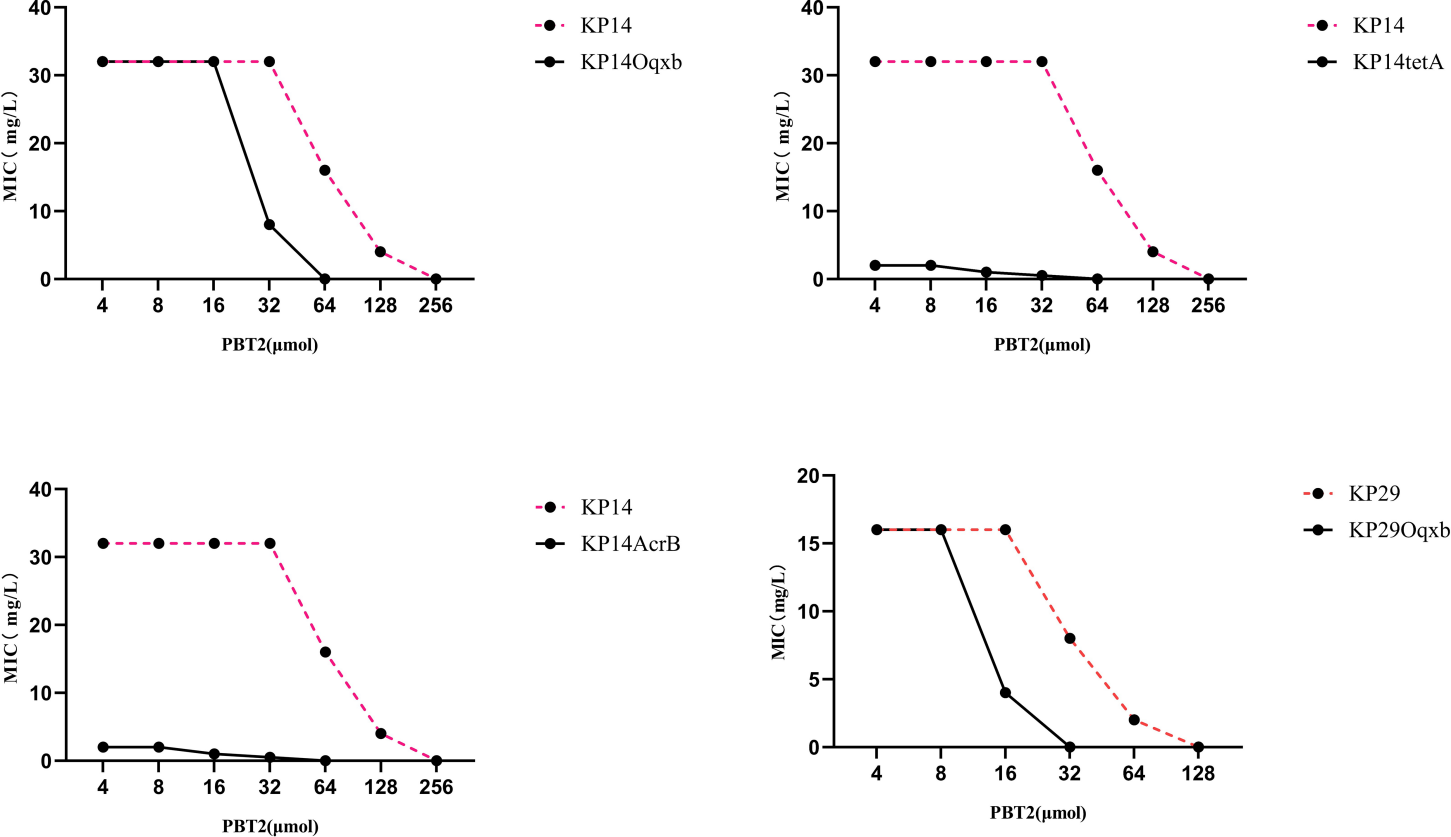
Changes in MIC values in KP29Oqxb, KP14Oqxb, KP14AcrB, and KP14tetA knockout strains. The MIC values of the four knockout strains were changed over 4-fold.

To further determine the factors influencing the changes in tigecycline resistance, we designed and constructed knockout strains of the efflux pump genes KP29Oqxb, KP14Oqxb, KP14AcrB, KP14tetA, and KP14AcrB-tetA, and conducted MIC experiments on these strains (**Figure 3**). The results indicated that the MIC values for the four strains KP29Oqxb, KP14Oqxb, KP14AcrB, and KP14tetA all changed by a factor of ≥4, and the KP14AcrB-tetA efflux pump gene knockout strain was unable to grow in culture medium containing tigecycline. Combined with the results in **Table 1**, this confirmed that the enrichment of zinc ions may, to some extent, limit the tigecycline resistance process dominated by the efflux pump mechanism.

## 3 Discussion

With the misuse and inappropriate use of antibiotics, as well as the slowing pace of antimicrobial drug development, treating multidrug-resistant bacteria has become an increasingly challenging global issue. Carbapenem-resistant *Klebsiella pneumoniae* (CRKP) has shown a year-on-year increase, further limiting drug options, with tigecycline and polymyxins becoming the last resort for CRKP infections. As the use of tigecycline in therapy increases, its resistance has garnered significant attention. Therefore, it is necessary to develop new antibiotics and identify new targets for treating resistant bacteria.

Initially developed for treating Alzheimer’s and Huntington’s diseases, PBT2 has drawn attention due to its unique zinc ion transport properties (Adlard et al., 2008). In the medical field, the combined use of PBT2 and zinc ions has been demonstrated to exhibit outstanding bactericidal effects against Gram-positive bacteria (Brazel et al., 2022; De Oliveira et al., 2022b).

In this study, we observed that the combined application of PBT2 and zinc ions significantly altered the multidrug resistance of CRKP to tetracycline antibiotics, especially tigecycline. This finding is consistent with research by De Oliveira, David M P et al (De Oliveira et al., 2020), whose experimental results indicated that the combined use of PBT2 and zinc ions not only altered CRKP’s resistance to tigecycline but also successfully reversed its resistance to polymyxins. In studies on Neisseria gonorrhoeae, PBT2 altered polymyxin resistance by reducing phosphoethanolamine modification of lipid A on the cell wall (Jen et al., 2020). However, there have been no previous reports on the specific mechanisms by which PBT2 affects tigecycline resistance. Therefore, building upon existing research, this study delves into the potential mechanisms underlying PBT2-mediated changes in tigecycline resistance.

The role of PBT2 in resistance is largely attributed to its ability to enrich zinc ions within cells. In this study, internal concentrations of iron, manganese, copper, and zinc ions in CRKP were measured using ICP-MS. The results confirmed that PBT2 significantly increased the intracellular zinc ion concentration (**Figure 1**). Excessive zinc ion concentrations may affect the uptake of iron and manganese ions to some extent. Early studies (Osman and Robinson, 2023) described complexes of divalent transition metals with polydentate acidic organic ligands, leading to the establishment of the Irving-Williams series: Mn²^+^ < Fe²^+^ < Co²^+^ < Ni²^+^ < Cu²^+^ > Zn²^+^. This series systematically reveals the variations in the thermodynamic stability of metal complexes: Mn and Fe exhibit relatively lower binding affinities, while Cu and Zn have higher binding affinities. Generally, metal ions with lower affinities can be easily replaced by ions with higher affinities. For example, manganese poisoning can lead to the inhibition of Mg-dependent enzymes (Hohle and O'Brian, 2014; Pi et al., 2020).Elevated zinc levels can impair manganese uptake through the mismetallation of Mn substrate-binding proteins (McDevitt et al., 2011).

CRKP exhibits diverse mechanisms of resistance to tigecycline, with the efflux pump transport system being the most predominant. These genes encode efflux pump proteins that anchor to the cell membrane and cell wall through a series of complex biological pathways. Bacteria pump out antimicrobial drugs through these efflux pumps to develop resistance. The tigecycline and PaβN combination experiment (**Supplementary Table S1**) confirmed that efflux pumps are the main factor contributing to tigecycline resistance. To explore the extent to which efflux pumps are involved in PBT2-induced changes in tigecycline resistance, this study utilized gene editing technology to construct CRKP strains with knockout mutations in the Oqxb, AcrB, TetA, and AcrB-TetA efflux pump genes. The CRKP strain with knockout mutations in the AcrB-TetA efflux pump genes was unable to grow in tigecycline-containing medium, and the MIC values of the other four strains increased by ≥4-fold (**Figure 3**). Importantly, the MIC values of meropenem did not change under PBT2 (64μM) and zinc ion (256μM) conditions. Combining these two sets of data suggests that intracellular zinc ion enrichment may limit tigecycline resistance primarily through efflux pump mechanisms. Transcriptomic studies further supported this hypothesis, showing upregulation of AcrB, Oqxb, RND and AcrD efflux pump genes in the PBT2+zinc treatment group(**Table 2**), although the upregulated efflux pump genes seemed ineffective in their function.

Based on recent research advances, we observed that PBT2 can alter MIC values, and the antimicrobial drugs affected by it appear to exhibit similarities in their resistance mechanisms. For example, PBT2 can reverse *Streptococcus pneumoniae* resistance to ampicillin (Brazel et al., 2022), as well as *Acinetobacter baumannii* and *Neisseria gonorrhoeae* resistance to tetracyclines (Jen et al., 2021; De Oliveira et al., 2022a), and *Streptococcus pyogenes* resistance to polymyxins (De Oliveira et al., 2022b). Most of these resistances are associated with changes in bacterial efflux pump mechanisms or binding sites on the cell wall. Further studies have revealed that zinc ion enrichment within cells significantly inhibits the acetyltransferase activity of GlmU enzyme, thereby severely affecting cell wall metabolism (Brazel et al., 2022). GlmU enzyme is a key bifunctional enzyme in cell wall synthesis responsible for UDP-GlcNAc synthesis, an essential precursor for cell wall biosynthesis. Our metabolomic data showed a downward trend in UDP-GlcNAc content in the PZinc group (**Supplementary Figure S3**). Additionally, the analysis of gene expression supported the notion that zinc ion enrichment may affect GlmU enzyme activity,GT2-GlmU-N-bac, UDPNAET, FosA, and GlmM are key genes involved in bacterial cell wall synthesis and their expression is upregulated (**Table 2**), although the upregulated genes seemed ineffective in their function. a hypothesis further validated by scanning electron microscopy observations. Building upon this research background and preliminary experimental results, we propose a potential resistance mechanism: PBT2, by promoting intracellular zinc ion enrichment in CRKP, affects the protein activity of GlmU enzyme, thereby reducing peptidoglycan synthesis. This process may also be accompanied by a loss of function of RND type efflux pumps at the cell membrane. The combined effects of these factors ultimately lead to the reversal of tigecycline resistance. However, how the blockage of bacterial cell wall synthesis pathways specifically affects the function of efflux pump proteins still requires further experimental validation. Meanwhile, considering the cost and toxicity of the PBT2 drug, animal experiments were not conducted in this study to assess its actual effects. Nevertheless, based on previous related research (Brazel et al., 2022; De Oliveira et al., 2022a), PBT2 combined with zinc ions has demonstrated favorable adjuvant therapeutic effects in mouse infection models.

## 4 Conclusion

Our study demonstrates that PBT2 effectively enriches intracellular zinc in *Klebsiella pneumoniae*, leading to the reversal of tigecycline resistance in a high-zinc environment. Moreover, this elevated zinc environment induces significant damage to K. pneumoniae’s biological systems, including growth metabolism and oxidative stress. Notably, disruption of zinc homeostasis markedly inhibits K. pneumoniae’s cell wall synthesis pathway. This inhibition may partially constrain the efflux pump mechanism, a primary driver of tigecycline resistance, thereby presenting novel perspectives and strategies for treating infections caused by this bacterium.

## 5 Experimental procedures

### 5.1 Efflux pump inhibition experiment

First, Mueller-Hinton (MH) agar medium with a pH between 7.2 and 7.4 was prepared. Different concentrations of tigecycline, appropriately diluted, were dissolved into the MH agar medium that had been heated to 45°C and mixed well. Then, the medium was allowed to cool to produce a thickness of 3-4mm. A bacterial suspension equivalent to the 0.5 McFarland standard turbidity was prepared and diluted 10 times for later use. A multipoint inoculator was used to inoculate 1-2μl of bacterial suspension onto the surface of the agar plates, which were then incubated at 35°C for 20 hours. The minimum inhibitory concentration (MIC) of the drug that inhibited bacterial growth was determined according to the methods recommended by the Clinical and Laboratory Standards Institute (CLSI).

### 5.2 Minimum inhibitory concentration determination

PBT2 powder was dissolved in DMSO solution using ultrasound for 10 minutes to prepare a 5 mM concentration solution, which was then stored at -80°C. A 20 mM solution of zinc sulfate, ferrous sulfate, copper sulfate, and manganese sulfate was prepared using deionized water and stored at room temperature. The minimum inhibitory concentrations (MIC) of antibiotics, PBT2, and zinc were determined using a slightly modified broth microdilution method. Zinc sulfate solution was added to cation-adjusted Mueller-Hinton broth (CAMHB) to achieve a final concentration of 256 μM zinc sulfate. Subsequently, the prepared CAMHB was divided into 8 groups, and PBT2 solution was added to each group to obtain solutions with different concentrations of PBT2 (256 μg, 128 μg, 64 μg, 32 μg, 16 μg, 8 μg, 4 μg, 0 μg). In the first column of a 96-well plate, the initial concentration of tigecycline solution (128 μg/ml) was added, and then serial dilutions were performed using the twofold dilution principle, discarding the last dilution. Finally, 100 μl of CAMHB containing bacterial inoculum was added to each well of the 96-well plate.

### 5.3 Development of resistance analyses

The KP29 strain was inoculated into CAMHB containing PBT2 and tigecycline at concentrations slightly below the MIC and cultured overnight. The next day, a small portion of well-grown culture was transferred to CAMHB containing higher concentrations of PBT2 and tigecycline (increased twofold). This process was repeated, with each transfer using culture from the previous day’s growth, until the strain could no longer grow in the presence of both PBT2 and tigecycline. Additionally, as a control, similar resistance development experiments were conducted using doxycycline.

### 5.4 Metal ion concentration detection

Bacterial cells were extracted from overnight cultures grown under different conditions, followed by centrifugation and washing. The collected bacteria were thoroughly dried using a desiccator and then weighed. Concentrated hydrochloric acid was added based on the weight to release the intracellular metal ions. The concentrations of iron, manganese, copper, and zinc in the four samples were determined using an ICP-MS instrument.

### 5.5 Growth curve experiment

Well-grown colonies were selected from blood agar plates and suspended in broth to achieve a 0.5 McFarland standard turbidity. Bacterial suspensions were then added to CAMHB, with or without pre-added PBT2 and zinc ions, and incubated with agitation. Portions of the culture were withdrawn at hourly intervals, and the optical density at 600 nm (OD600nm) was measured using a spectrophotometer.

### 5.6 Scanning electron microscopy experiment

Cultured bacteria were collected, washed several times with PBS (pH 7.0), and resuspended in clean bacterial pellets. The pellets were fixed in 2.5% glutaraldehyde solution at 4°C for 10 hours, followed by centrifugation and washing with PBS. Dehydration was performed using a series of ethanol solutions (10%, 30%, 50%, 70%, 80%, 90%, 100%) for 5 minutes each. The samples were then dried with hexamethyldisilazane for 20 minutes and coated with gold using a high-vacuum ion sputtering instrument at 30 mA for 30 seconds. Observation was conducted under a field emission scanning electron microscope.

### 5.7 Detection of H2O2 content and SOD enzyme activity in K. pneumoniae under different conditions

The well-grown bacteria were collected into centrifuge tubes, and the supernatant was obtained. According to the standard procedure, 1 ml of acetone extraction solution was added per 5 million bacteria, followed by sonication (power 200 W, 3 seconds on, 10 seconds off, repeated 30 times). The mixture was then centrifuged, and the supernatant was collected. The corresponding assay kit (Shanghai Sangon Biotech Co., Ltd.) was used according to the manufacturer’s instructions.

### 5.8 Metabolomics experiments

Bacteria cultured with different drugs were harvested at OD600nm=1.5, and corresponding bacterial samples were collected. An appropriate amount of sample was added to pre-cooled methanol/acetonitrile/water solution (2:2:1, v/v), vortexed, sonicated for 30 min at low temperature, incubated for 10 min, centrifuged at 14000 g for 20 min at 4°C, and the supernatant was vacuum-dried. Then, 100 μL of acetonitrile-water solution (acetonitrile=1:1, v/v) was added, vortexed, centrifuged at 14000 g for 15 min at 4°C, and the supernatant was taken for analysis. Samples were separated using an Agilent 1290 Infinity LC ultra-high performance liquid chromatography system (UHPLC) and analyzed by Triple TOF 6600 mass spectrometer (AB SCIEX) for metabolite detection in both positive and negative ion modes using electrospray ionization (ESI). Bacterial RNA was extracted using the Bacterial RNA Rapid Extraction Kit (Shanghai Sangon Biotech Co., Ltd.) for transcriptomics analysis. Raw sequencing data were quality assessed using FastQC, quality-trimmed using Trimmomatic to obtain reliable data. Rockhopper was used for *de novo* transcriptome assembly, and various information of the assembled transcripts was summarized.

### 5.9 Gene knockout experiment

According to the outer membrane pump gene sequences reported by NCBI, gRNAs were designed online using (http://crispor.tefor.net/crispor.py). The plasmid pet41a was used as an expression vector, and the plasmid pwt-Cas9 containing the Cas9 gene sequence was obtained to construct the recombinant plasmid pet41a-Cas9. The vector PET41a and pwt-Cas9 were digested with XhoI and BglII restriction endonucleases, and the digested products were subjected to 37°C water bath for 3 h, followed by agarose gel electrophoresis. The digested gene products were recovered, ligated, and transformed into Escherichia coli DH5α competent cells by heat shock method overnight. The transformed cells were spread on LB solid medium containing antibiotics for colony selection. PCR-positive clones of the recombinant plasmid were screened, and the products were sequenced. The ligated products were transformed into Escherichia coli DH5α competent cells, and positive clones were screened by PCR and confirmed by SDS-PAGE protein gel electrophoresis, detecting proteins with molecular weights matching the theoretical value of Cas9 protein. Thus, successful construction of the recombinant expression system was confirmed. The recombinant plasmid was introduced into the target strain by electroporation or phage transformation, followed by overnight incubation at 30°C. Drug sensitivity tests were performed on the constructed gene knockout strains to observe changes in the MIC values of tigecycline, and real-time fluorescence quantitative PCR experiments were performed to detect changes in the expression levels of outer membrane pump genes.

### 5.10 Transcriptomics experiments

Bacterial RNA was extracted using the Bacterial RNA Rapid Extraction Kit (Shanghai Sangon Biotech Co., Ltd.) for transcriptomics analysis. 1ml of bacterial solution in the logarithmic growth phase was taken, centrifuged, and washed several times. Then, 20 μl of lysozyme was added for lysis. The lysed sample or homogenate was allowed to sit at room temperature for 5-10 minutes to ensure complete separation of nucleoproteins and nucleic acids. Next, 0.2 ml of chloroform was added, and the mixture was shaken vigorously for 15 seconds before being left at room temperature for 3 minutes. It was then centrifuged at 12,000 rpm at 4°C for 10 minutes. The upper aqueous phase was transferred to a clean centrifuge tube, an equal volume of isopropanol was added, and the mixture was well mixed and left at room temperature for 20 minutes. After that, it was centrifuged at 12,000 rpm at 4°C for 10 minutes, and the supernatant was discarded. The precipitate was washed with 1 ml of 75% ethanol, centrifuged at 12,000 rpm at 4°C for 3 minutes, and the supernatant was again discarded. The precipitate was allowed to dry at room temperature for 5-10 minutes. Subsequently, 30-50 μl of RNase-free ddH2O was added to fully dissolve the RNA. The obtained RNA solution was stored at -70°C. The subsequent steps, including rRNA removal, RNA fragmentation, reverse transcription, library construction, sequencing, and data analysis, were handled by Shanghai Sangon Biotech Co., Ltd.

## Data Availability

The original contributions presented in the study are publicly available. This data can be found here: NCBI, PRJNA1219305.
