# Supplementary material for: Disruption of zinc homeostasis reverses tigecycline resistance in Klebsiella pneumoniae
Source: Front Cell Infect Microbiol. 2025 Feb 12;15:1458945. doi: 10.3389/fcimb.2025.1458945 (PMC11860891; doi:10.3389/fcimb.2025.1458945)
Supplement: Supplementary file 1 [file Table1.docx]

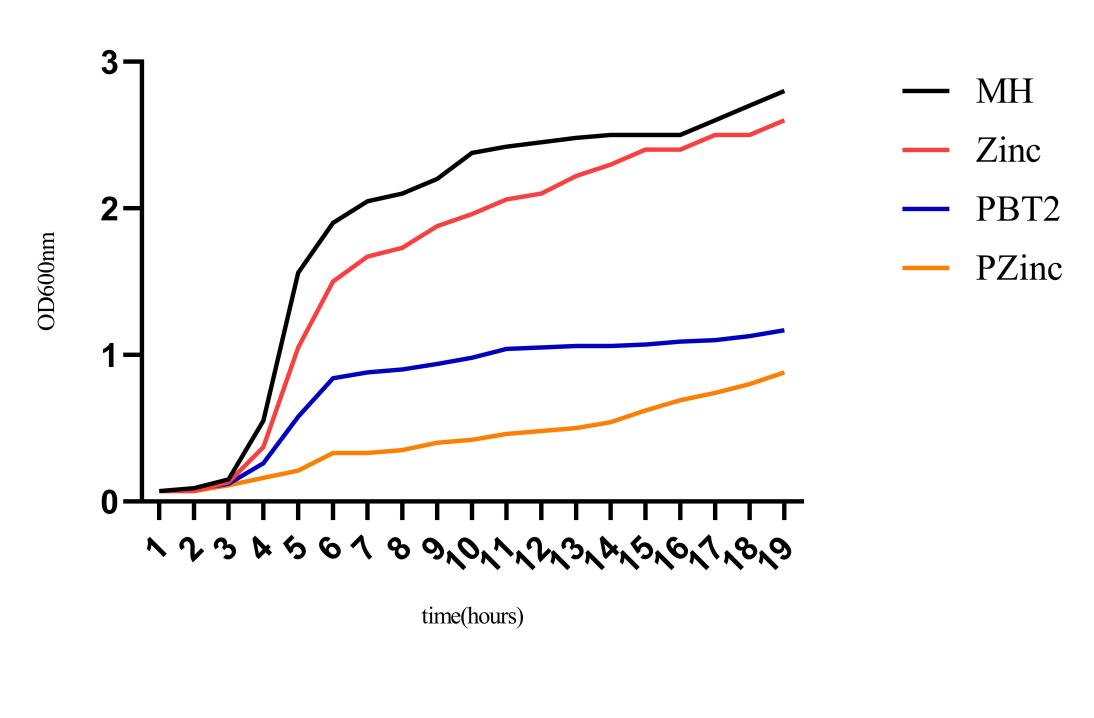


Figure S1: Changes in growth curves of K. pneumoniae upon application of PBT 2 and zinc ion. Data represent the means from two biological replicates.

Figure S2: altered resistance of K. pneumoniae in response to PBT 2 + Zinc. Data are representative of three biological replicates.


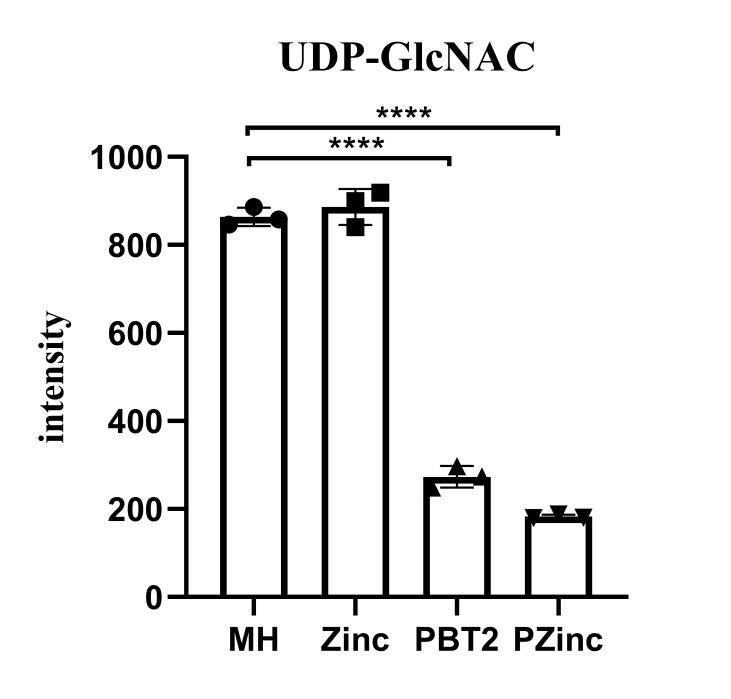


Figure S3: Results of internal UDP-GlcNAc content of K. pneumoniae under different conditions. Error bars represent SD from three biological replicates.


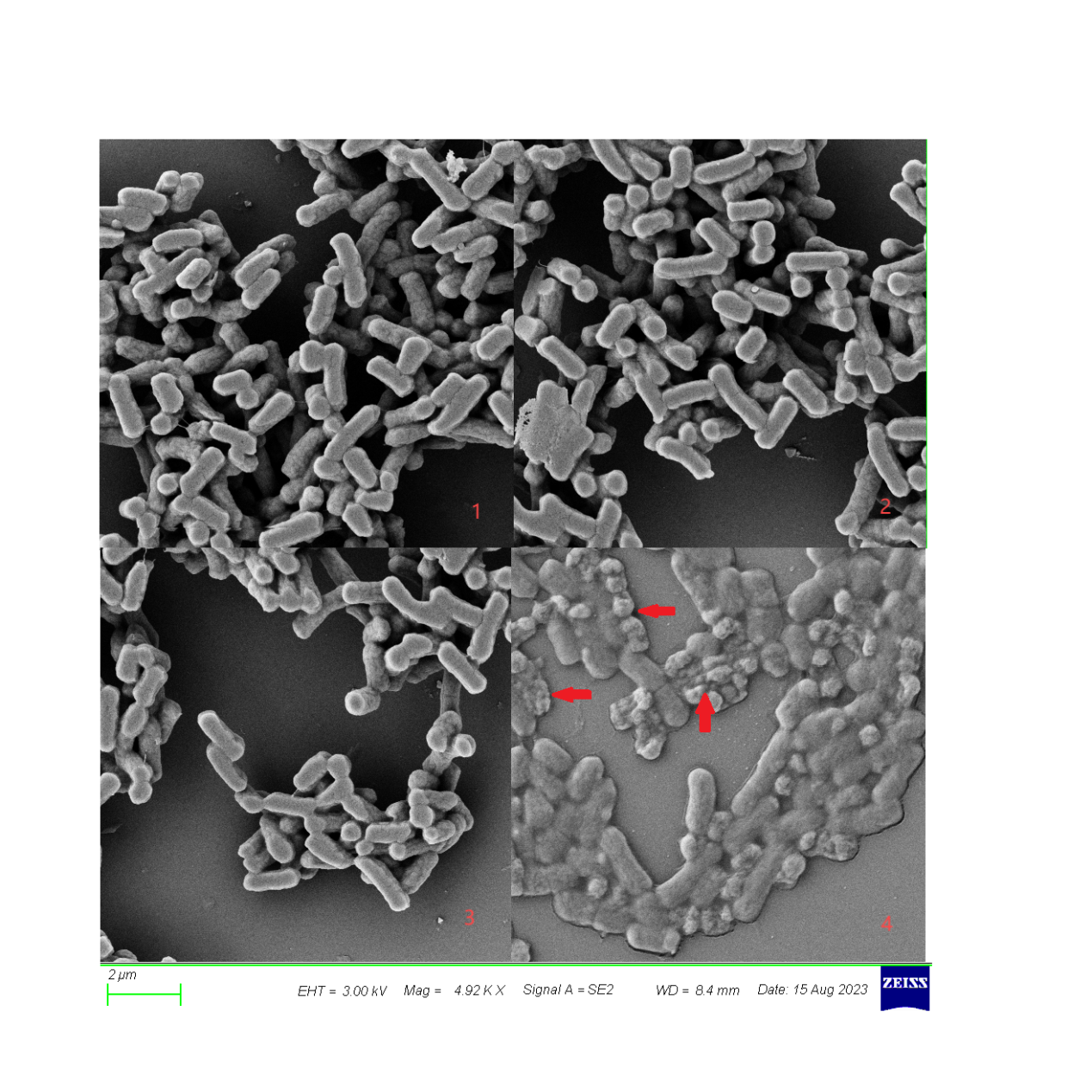


Figure S4: Changes in cell morphology of Klebsiella pneumoniae under SEM (1. MH group, 2. Zinc group, 3. PBT 2 group, 4. PZinc group. Arrows indicate membrane indentations and membrane ruffling). Scale bars = 2 μm.

Table S1. Results of tigecycline combined with efflux pump inhibitors

| **Isolates** | **TGC (μg/mL)** | **TGC+****PAβN (μg/mL)** |
| --- | --- | --- |
| ATCC13883 | 0.125 | 0.125 |
| KP9 | 4 | 1 |
| KP10 | 8 | 2 |
| KP14 | 32 | 4 |
| KP16 | 16 | 2 |
| KP17 | 8 | 2 |
| KP18 | 16 | 2 |
| KP21 | 4 | 1 |
| KP23 | 8 | 2 |
| KP25 | 4 | 1 |
| KP28 | 4 | 1 |
| KP29 | 16 | 2 |
| KP30 | 8 | 1 |
| KP32 | 8 | 2 |
| KP56 | 8 | 1 |
